# Memory extinction and spontaneous recovery shaping parasitoid foraging behavior

**DOI:** 10.1093/beheco/arab066

**Published:** 2021-06-30

**Authors:** Jessica A C de Bruijn, Louise E M Vet, Hans M Smid, Jetske G de Boer

**Affiliations:** 1 Laboratory of Entomology, Plant Sciences Group, Wageningen University, Droevendaalsesteeg 1, 6708 PB Wageningen, The Netherlands; 2 Department of Terrestrial Ecology, Netherlands Institute of Ecology (NIOO-KNAW), Droevendaalsesteeg 10, 6708 PB Wageningen, The Netherlands

**Keywords:** context-specificity, optimal foraging behavior, memory formation, parasitoid wasp, plant species, unrewarding experience

## Abstract

Animals can alter their foraging behavior through associative learning, where an encounter with an essential resource (e.g., food or a reproductive opportunity) is associated with nearby environmental cues (e.g., volatiles). This can subsequently improve the animal’s foraging efficiency. However, when these associated cues are encountered again, the anticipated resource is not always present. Such an unrewarding experience, also called a memory-extinction experience, can change an animal’s response to the associated cues. Although some studies are available on the mechanisms of this process, they rarely focus on cues and rewards that are relevant in an animal’s natural habitat. In this study, we tested the effect of different types of ecologically relevant memory-extinction experiences on the conditioned plant volatile preferences of the parasitic wasp *Cotesia glomerata* that uses these cues to locate its caterpillar hosts. These extinction experiences consisted of contact with only host traces (frass and silk), contact with nonhost traces, or oviposition in a nonhost near host traces, on the conditioned plant species. Our results show that the lack of oviposition, after contacting host traces, led to the temporary alteration of the conditioned plant volatile preference in *C. glomerata,* but this effect was plant species-specific. These results provide novel insights into how ecologically relevant memory-extinction experiences can fine-tune an animal’s foraging behavior. This fine-tuning of learned behavior can be beneficial when the lack of finding a resource accurately predicts current, but not future foraging opportunities. Such continuous reevaluation of obtained information helps animals to prevent maladaptive foraging behavior.

## Teaser

The parasitic wasp Cotesia glomerata temporarily ceases to respond to learned plant volatiles when the expected host caterpillar is not found, but only its traces, such as frass and silk. Such fine-tuning of learned behavior can be beneficial when the lack of host finding accurately predicts current, but not future opportunities. In this way, animals continuously re-evaluate available information to prevent maladaptive foraging behavior.

## INTRODUCTION

Foraging for resources in a complex environment is a demanding task for animals. Natural environments are highly complex and variable in time and space, which has led to the evolution of behavioral adaptations to optimize foraging efficiency (Raine et al. 2006; Hilker and McNeil 2008; Eliassen et al. 2009). One such adaptation is associative learning, where an encounter with a resource is associated with the perception of nearby environmental cues, such as the classical example of the sound of a bell signaling food for Pavlov’s dogs (Clark 2004), but also bees associating volatiles and colors of flowers with nectar (Hazlett 1994; Dukas 1999a; Menzel 1999; Eisenhardt 2012; Haverkamp and Smid 2020). These kinds of rewarding experiences can lead to the formation of an associative reward memory, which alters foraging behavior, and can improve foraging efficiency (Macphail 1996; Durier and Rivault 2000; Raine and Chittka 2008; Hoedjes et al. 2011; Warburton and Hughes 2011; Zrelec et al. 2013). However, the reliability of the obtained information, that is it correctly predicting resource presence, is likely to be highly variable. Extensive theoretical studies exist on how animals should deal with information reliability (Speed and Turner 1999; Koops 2004; Dall et al. 2005; Dunlap and Stephens 2012, 2016) and a wealth of studies on (associative) learning and cognitive abilities exist (Dukas 2004; Mery 2013; Smid and Vet 2016). However, how information reliability shapes animal foraging behavior in the context of associative learning is not well understood and studies using information and resources that are relevant in an animal’s natural habitat are uncommon.

The reliability of associative memory is reevaluated on subsequent encounters with the associated cues during foraging. A second encounter with the resources, after perceiving and responding to the associated cues, will strengthen the memory and enhance the future response to the associated cues (Lee 2008). However, animals may also be led to patches containing only resource traces or even unsuitable resources. For example, after associating yellow with nectar presence, bees may be attracted to yellow flowers that no longer contain nectar (Eisenhardt 2012). When expected resources are not encountered on responding to learned cues, a memory-extinction experience occurs, that is the animal learns that the associated cues do not always reliably predict resource presence (Bouton 2004). This experience can induce the formation of so-called extinction memory, that exists in parallel and suppresses recall of the earlier formed memory, rather than that it erases that memory. Thus, the original memory is still intact but the extinction memory suppresses its behavioral expression. Memory extinction is therefore a form of active (temporary) forgetting that is different from passive forgetting through natural memory decay, which occurs when memory is no longer intact after a certain time (Davis and Zhong 2017, Piqueret et al. 2019). After the extinction memory has waned, recall of the original memory is no longer suppressed and the conditioned behavior can be expressed again. This phenomenon is called spontaneous recovery (see below). The preservation of this unreliable memory and the continuing use of the unreliable information can be costly, but it can still have a net benefit when the information is only rarely unreliable. Even when the information is frequently unreliable, it can be worthwhile when the cost of using the information is relatively small compared with the benefit when the information proves to be reliable (Koops 2004).

At the brain level, behavioral change due to unreliable information depends on the strength of the reward memory, and the strength and timing of the extinction experience. This has been shown in vertebrates (for example Lee 2008; Exton-McGuinness et al. 2015; Harris and Andrew 2017), but also in insects, such as fruit flies (for example Lagasse et al. 2009) and bees (for example Eisenhardt 2012; Eisenhardt et al. 2013). The ease and speed with which behavior is altered through memory processes, is part of an animal’s preparedness to learn, which is shaped by the animal’s ecology (Kruidhof et al. 2012; Dunlap and Stephens 2016; Smid and Vet 2016). The strength of the associative reward memory is influenced by the value of the reward, the expected reliability of the information, and subsequent encounters of the same combination of cue and associated reward (Lee 2008). Together, these aspects determine the memory stages that are formed (short-, mid-, and long-term memory) and their temporal dynamics (consolidation speed and persistence) (Honig and James 1971; Smid and Vet 2016). The same processes apply to the formation of the extinction memory (Eisenhardt 2012; Exton-McGuinness et al. 2015). When the strength of the extinction memory outweighs the strength of the reward memory, the retrieval of the reward memory is inhibited and the conditioned behavior is no longer observed (Suzuki et al. 2004; Stollhoff et al. 2005). In addition, the effect of an extinction experience depends on the timing of the extinction experience relative to the reward memory formation. Over time, a rewarding experience is stored in different memory stages, with each subsequent memory stage more resistant to the effects of an extinction experience (Lagasse et al. 2009; Eisenhardt 2012; Miguez et al. 2012). Together, the reward memory and the extinction experience either cause no change in conditioned behavior (strong reward memory and/or weak extinction experience) or permanently change the conditioned behavior (the opposite) (Ecker 2015; Exton-McGuinness et al. 2015). An intermediate scenario may occur when the conditioned behavior is temporarily altered due to the formation of an extinction memory that decays faster than the reward memory, resulting in the spontaneous recovery of the conditioned behavior (Stollhoff et al. 2005).

The mechanistic understanding of the effect of extinction experiences is largely based on laboratory experiments performed with unnatural (artificial) cues and rewards or punishments (Kaiser et al. 2003; Lagasse et al. 2009; Eisenhardt et al. 2013; Harris and Andrew 2017). For example, Harris and Andrew (2017) investigated the effect of the number and duration of extinction experiences in rats with artificial food pellets and artificial light and sound. It is questionable whether animal foraging behavior is affected in the same way when natural environmental cues and rewards are used because artificial cues and rewards lack natural variation and are not necessarily relevant in an ecological context. We address this topic here by studying how extinction experiences with natural cues and rewards influence the foraging behavior of parasitoid wasps.

Parasitoids lay their eggs in or on the bodies of other (host) insects, which their offspring eventually kill (Godfray 1994). Parasitoid host searching behavior is under strong selection pressure, because it is directly related to offspring production and therefore to fitness. It is well known that parasitoids use environmental cues to locate their inconspicuous hosts (Vet et al. 1991), for example, herbivore-induced plant volatiles (HIPVs). Associative learning of HIPVs and memory formation have been studied extensively in these insects (Kaiser et al. 2003; Bleeker et al. 2006; Costa et al. 2010; Hoedjes et al. 2011; Kruidhof et al. 2015; Smid and Vet 2016; de Bruijn et al. 2018; Vosteen et al. 2019; Haverkamp and Smid 2020). We expect that oviposition-related memories are relatively resistant to extinction experiences because of the strong link between oviposition and parasitoid fitness (Papaj and Vet 1990; Kruidhof et al. 2015).We, therefore, hypothesize that extinction experiences only lead to temporary suppression followed by spontaneous recovery of the conditioned host-searching behavior of parasitoids, or even to no changes at all.

In nature, parasitoids may encounter different types of memory extinction experiences. HIPVs previously associated with a host oviposition might lead them to plants with nonhosts (that is insect species that are not suitable for the development of parasitoid offspring), or to traces (for example frass and silk) of a host or nonhost that is no longer present. We expect that an extinction experience with host traces does not lead to changes in behavior, because host traces are tightly connected with host presence, and contact with host traces can itself lead to the formation of reward memory in parasitoids (Hoedjes et al. 2011). Instead of representing an extinction experience, encountering host traces may therefore rather strengthen the reward memory, by confirming the association between HIPVs and hosts, despite their physical absence. In contrast, we expect that an extinction experience with nonhosts and their traces will lead to the formation of extinction memory, especially when an associative reward memory is not fully consolidated yet because nonhost related encounters clearly indicate that the learned information does not reliably predict host presence.

To test these hypotheses, we used *Cotesia glomerata*, a larval endoparasitoid of caterpillars of the large cabbage white *Pieris brassicae* (Laing and Levin 1982) and the nonhost caterpillar *Mamestra brassicae*, which feeds on the same plant species and has a very similar life history (Bell and Muller 1973; Carter 1984). *Mamestra brassicae* caterpillars are unsuitable for *C. glomerata* offspring development but accepted for oviposition (de Bruijn et al. 2018, de Bruijn et al. manuscript in preparation). *C. glomerata* has an innate response to HIPVs of certain plant species, but its preference can be altered through associative learning of HIPVs of other plant species (Geervliet et al. 1996, 1998). A single oviposition in the *P. brassicae* caterpillar host can already result in the formation of long-term memory (Smid et al. 2007) and we used this type of single trial conditioning in this study. Ten minutes thereafter, we exposed *C. glomerata* to different types of extinction experiences, when short-term memory is present but long-term memory is not consolidated yet (van den Berg et al. 2011), to test for the disappearance and spontaneous recovery of the conditioned plant volatile preference. Furthermore, we tested whether these extinction experiences can lead to plant species-specific results, using two closely related Brassica species: black mustard (*Brassica nigra*) and red cabbage (*Brassica oleracea* var. rubra). These closely related brassicaceous plant species were selected because previous work (Geervliet et al. 1998; de Bruijn et al. 2018) showed that *C. glomerata* can learn to associate their HIPVs with host presence using a single conditioning trial.

## MATERIALS AND METHODS

### Insects

All insect colonies were reared at the Laboratory of Entomology, Wageningen University. Insects were kept in a greenhouse with a photoperiod of L16:D8 (both natural and artificial light), at 21 ± 1 °C and 50–70% relative humidity. Colonies were reestablished each year with individuals collected from cabbage fields around Wageningen, the Netherlands. *Cotesia glomerata* (Hymenoptera: Braconidae) was reared on first instar caterpillars of *Pieris brassicae* (Lepidoptera: Pieridae). Caterpillars of both *P. brassicae* and the nonhost species *Mamestra brassicae* were reared on Brussels sprouts plants (*Brassica oleracea* L. var. gemmifera cultivar Cyrus).

Newly formed *C. glomerata* cocoons were collected from the colony and transferred to mesh cages in groups of approximately 300 individuals (30 × 30 × 30 cm, Bugdorm-1 Insect rearing cage, type DP1000, Megaview Science, Taiwan). These cages were placed in a climate chamber set at 21 ± 1 °C, 50–70% relative humidity, and a photoperiod of L16:D8. Emerging males and females were provided with water and honey and could freely mate. Two days after adult emergence, approximately 100 females were transferred to a clean cage with water and honey and placed in the same climate chamber. For experiments, unconditioned females were used 3–5 days after adult emergence, that is females without any prior experience with plants, hosts, nonhosts and their traces.

### Plant induction

We used black mustard (*Brassica nigra*) and red cabbage (*Brassica oleracea* var. rubra (DC) cv. Langedijker bewaar) plants in experiments when they were four weeks old. Plants were kept in a climate-controlled greenhouse at 21 ± 1 °C, 50–70% relative humidity and a L16:D8 photoperiod. To induce emission of HIPVs, plants were infested 24 h before use with either 1^st^ instar host caterpillars (*P. brassicae*) or 1^st^ instar nonhost caterpillars (*M. brassicae*). Plants used to test plant volatile preference were infested by placing a group of 20 host caterpillars on the third leaf of a black mustard or red cabbage plant. Plants used for conditioning were infested with a group of approximately 50 host caterpillars on each of the four youngest fully expanded leaves. Black mustard and red cabbage leaves used during the extinction phase were infested with either a group of 30 host caterpillars or 30 non-host caterpillars, placed on the two youngest fully expanded leaves of a plant. Because the nonhost caterpillars often dropped from the plant, they were placed in two clip cages, each with 15 nonhost caterpillars, which were attached to a single leaf. The use of these induced plants in experiments is described below.

### Parasitoid conditioning

#### Extinction experiment

**Figure 1:**
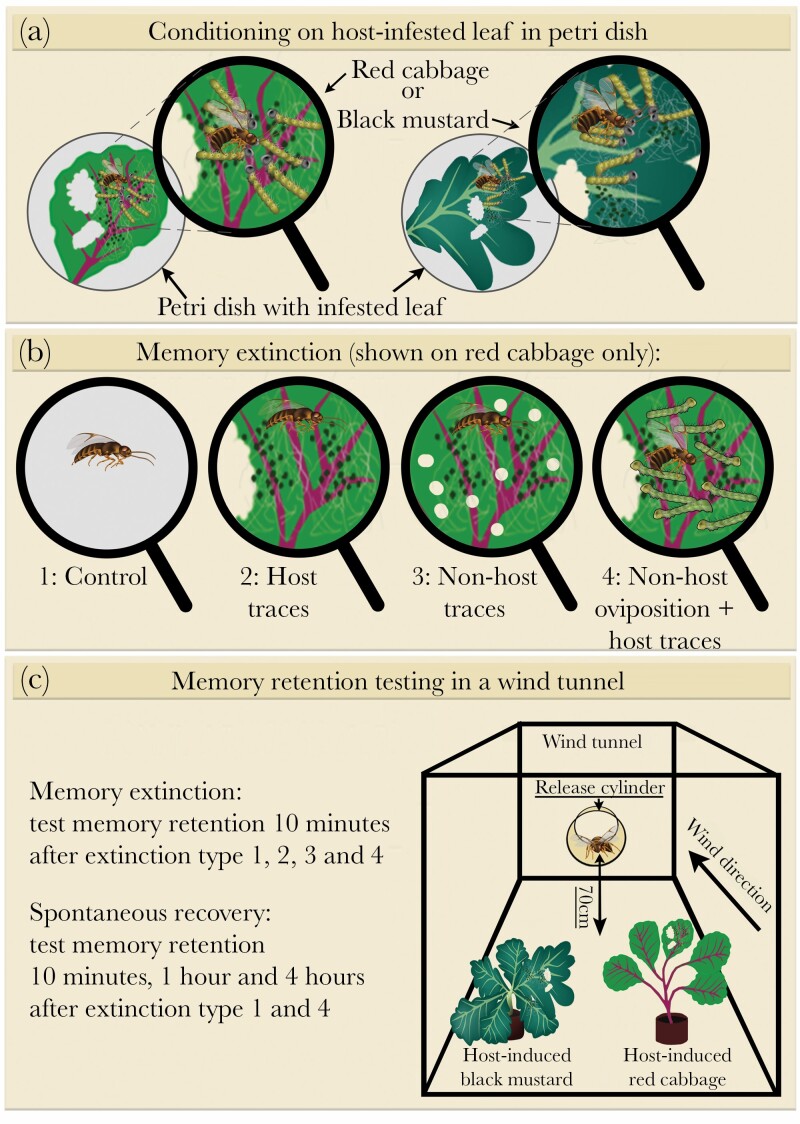
Schematic representation of the experimental approach. The enlarged view, represented by a looking glass, shows all components of the conditioning: the presence of the parasitoid, *P. brassicae* host caterpillars (yellow with black head), *M. brassicae* nonhost caterpillars (green with yellow head), silk and frass as host traces (white strands and green dots) and feeding damage (holes in the leaf surface). (a) Conditioning of the wasps on a host-infested leaf of either red cabbage or black mustard in a Petri dish. (b) The 4 different extinction treatments occurred in a Petri dish on a leaf of the conditioned plant species 10 min after conditioning: (1) control treatment in an empty Petri dish; (2) experiencing host traces; (3) experiencing nonhost traces (note the difference in feeding patterns of the solitarily-feeding nonhosts *M. brassicae* and the gregariously-feeding hosts *P. brassicae* caterpillars); (4) oviposition in a nonhost placed on a leaf with host traces. (c) Testing memory retention in a wind tunnel. The wind tunnel (right) is shown in a frontal view, with the plants with host feeding damage placed upwind, and the release cylinder with a wasp placed 70 cm downwind of the plants. Each wasp was tested only once. The same setup was used for testing the extinction and spontaneous recovery experiment.

The extinction experiment consisted of 3 phases; 1) the conditioning phase, 2) the extinction treatments; 3) testing parasitoid preference in the wind tunnel (Figure 1). In phase 1, four parasitoids were individually conditioned by allowing each parasitoid to oviposit once in a host caterpillar on either a black mustard or red cabbage leaf, thereby giving them a rewarding experience. This leaf was detached from the host-infested plant just before conditioning. After observing one oviposition, the parasitoid was gently removed from the leaf and subsequently placed in a mesh cage (17.5 × 17.5 × 17.5 cm, Bugdorm-41515 Insect Cage, type BD41515, Megaview Science, Taiwan) with 3 other parasitoids that were conditioned on the same leaf. They were kept in this cage with water and honey for 10 min. Parasitized caterpillars were removed directly after the oviposition to prevent superparasitism.

In phase 2, these 4 conditioned parasitoids were then each given a different extinction treatment. Three parasitoids were given an extinction experience on a leaf of the same plant species as used for conditioning, although one served as a positive control, being handled the same way, but not exposed to any plant and (non)host material. The four extinction treatments, therefore, consisted of: 1) a control (C), where the parasitoid was directly placed in an empty Petri dish (94 × 16 mm, Greiner bio-one, Germany) for 15 min; 2) host traces (HT), where the parasitoid was exposed to frass and silk of its host on a leaf in a Petri dish for 15 min; 3) nonhost traces (NHT), where the parasitoid was exposed to frass and silk of the non-host on a leaf in a Petri dish for 15 min; 4) nonhost oviposition (NHO), which consisted of two steps, first the parasitoid was given a single oviposition in a nonhost caterpillar on a leaf with host traces in a Petri dish. After that, the parasitoid was placed in an empty Petri dish for 15 min. Placement of the parasitoid in an empty Petri dish in the control (C) and nonhost oviposition (NHO) extinction treatments was done to standardize phase 2 procedures, in terms of handling and the time spent in the confined space of the Petri dish. After the extinction treatment, parasitoids were individually transferred to cages with water and honey for 10 min before being tested in phase 3 (see below in Section Wind tunnel assay). Parasitoids were used once and then discarded.

Both the conditioning and the extinction experience occurred on a leaf in a closed Petri dish to limit the emanation of HIPVs into the laboratory and to keep the leaves fresh for a longer period. Petri dishes were kept closed at all times, except for a short moment when the parasitoid was introduced. Leaves were detached from the plant just before their use. In the conditioning phase, one leaf was used for a single group of 4 parasitoids, after which it was discarded. In the extinction phase, two leaves with host traces (for the host traces and nonhost oviposition treatments) and one with nonhost traces (for the nonhost traces treatment) were used. Caterpillars were removed before placing each leaf in its own Petri dish and leaves were discarded after a single use. We placed a small (approximately 0.8 cm) moist cotton ball in empty Petri dishes in the control and nonhost oviposition treatments to reduce the static properties of the Petri dishes and elevate relative humidity, to mimic conditions of Petri dishes with a leaf.

In total there were 8 treatments, 4 extinction treatments on each of 2 plant species. One run consisted of 4 parasitoids (1 per extinction treatment for a given conditioned plant species) completing the three phases of the extinction experiment. Thereafter, a new run, executed in the same manner, was started. Each day, 8 runs were completed, 4 consecutive runs with conditioning and extinction on black mustard followed by 4 consecutive runs with conditioning and extinction on red cabbage, or the other way around. We executed the extinction experiment on 15 days and randomized the order of the two plant species among days. Because some parasitoids were lost during the process (for example escaped), 45–56 individual parasitoids were tested for each treatment.

#### Spontaneous recovery experiment

For the spontaneous recovery experiment, an experimental run was designed in the same way as described above. Based on the results of the extinction experiment, the spontaneous recovery experiment was executed only with black mustard as plant species for conditioning and extinction, and with only two of the extinction treatments described above: control (C) and host traces (HT). Phase 3 was slightly different compared with the extinction experiment because wind tunnel tests were performed directly after the extinction phase (that is 10 min after the extinction treatment), 1 h and 4 h after the extinction phase to test for spontaneous recovery of the conditioned plant volatile preference. Individual parasitoids were tested only at one time point.

Per run, we used 12 parasitoids, 2 for each of the 6 treatments (2 extinction treatments * 3 time points). As described above in phase 1, all 12 parasitoids were first individually given a rewarding experience on black mustard in a Petri dish, and subsequently, they were housed together in a cage for 10 min with water and honey. In phase 2, 6 of these 12 parasitoids were given an extinction treatment with host traces on a black mustard leaf in a Petri dish for 15 min. The other 6 parasitoids were assigned to the control treatment, where they were individually placed in an empty Petri dish for 15 min. As a preparation for phase 3, the 12 parasitoids were transferred to 6 clean cages with water and honey, 3 cages for parasitoids of the host traces extinction treatment, and 3 for the control parasitoids. Each of these 3 cages was assigned to one of the 3 time points (10 min, 1 h or 4 h) and housed 2 parasitoids. In phase 3, we tested the plant species preference of 4 parasitoids at each time point, 2 parasitoids of the control and 2 of the host traces extinction treatment.

We conducted 3 runs per day. Due to the long waiting time between the different time points, a subsequent run was started after testing parasitoids of the previous run at the 10 min time point. We executed the spontaneous recovery experiment on 9 experimental days, with 36 parasitoids per day (*n* = 6 per treatment per day). Due to some losses during the process, a total of 41–47 parasitoids per treatment was tested.

### Wind tunnel assay

The olfactory response of parasitoids was tested in a wind tunnel as described by Geervliet et al. (1994) and depicted in Figure 1c, at 24 ± 1 °C with a relative humidity of 50–70% and a wind speed of 10 cm/s. Two test plants, a host-induced black mustard, and a host-induced red cabbage plant, were placed 70 cm upwind from the release point, 30 cm from each other, and 10 cm from the walls of the wind tunnel.

For each test, a single parasitoid was released in the center of the glass release cylinder (30 cm long, 15 cm diameter). The parasitoid was given 5 min to depart from the release cylinder and land on an induced plant. Once landed, its choice was recorded and it was removed from the wind tunnel. Parasitoids that directly flew to the wind tunnel ceiling on departure from the release cylinder, were recaptured and placed in the glass release cylinder once more. Parasitoids that did not land on an induced plant within 5 min were removed from the experiment.

Test plants were used for a maximum of 4 h, except in the spontaneous recovery experiment, where we used a new set of plants for each time point, to minimize effects of decreased plant volatile release over time.

### Statistical analysis

All statistical analyses were done in R 3.5.0. To test for plant volatile preference of parasitoids within a certain treatment, the total number of parasitoids choosing either plant species was determined and a binomial test was used to evaluate if this distribution differed from a 50:50 distribution over the two plant species. To evaluate treatment effects, that is effects of conditioning on different plant species, effects of the extinction treatments and time points, on parasitoid plant volatile preference (choice), we used full factorial generalized linear mixed models (GLMMs) with a Bernoulli distribution and a Logit link function. For the extinction experiment, we used plant species, extinction treatment, and their interaction as fixed effect terms and run number as a random effect term. The model for the spontaneous recovery experiment included extinction treatment, time (continuous), and their interaction as fixed effect terms and day as a random effect term. To evaluate that the control group maintained memory at all time points in the spontaneous recovery experiment, an additional model was used with a subset of data of the control parasitoids at different time points. The model included time as a fixed effect term and day as a random effect term. Random effect terms were selected for each model based on the lowest AIC score (highest added value). All models were checked for overdispersion. Interaction terms were retained in the models because they had (marginally) significant effects in the extinction and spontaneous recovery experiment (see below).

For the extinction model, we used the glmer function from the lme4 package (Bates et al. 2014). We chose the mixed_model function from the package GLMMadaptive (Rizopoulos 2019) for the models in the spontaneous recovery experiment, because it is better suited for binomial GLMMs with a continuous fixed effect. These models used the integration method adaptive Gauss-Hermite quadrature rule and the optimization method hybrid EM and quasi-Newton. Post hoc testing was performed with the lsmeans function with adjusted Sigma (Tukey’s test) of the lsmeans package (Lenth 2016).

## RESULTS

### The effect of different types of extinction experiences

**Figure 2:**
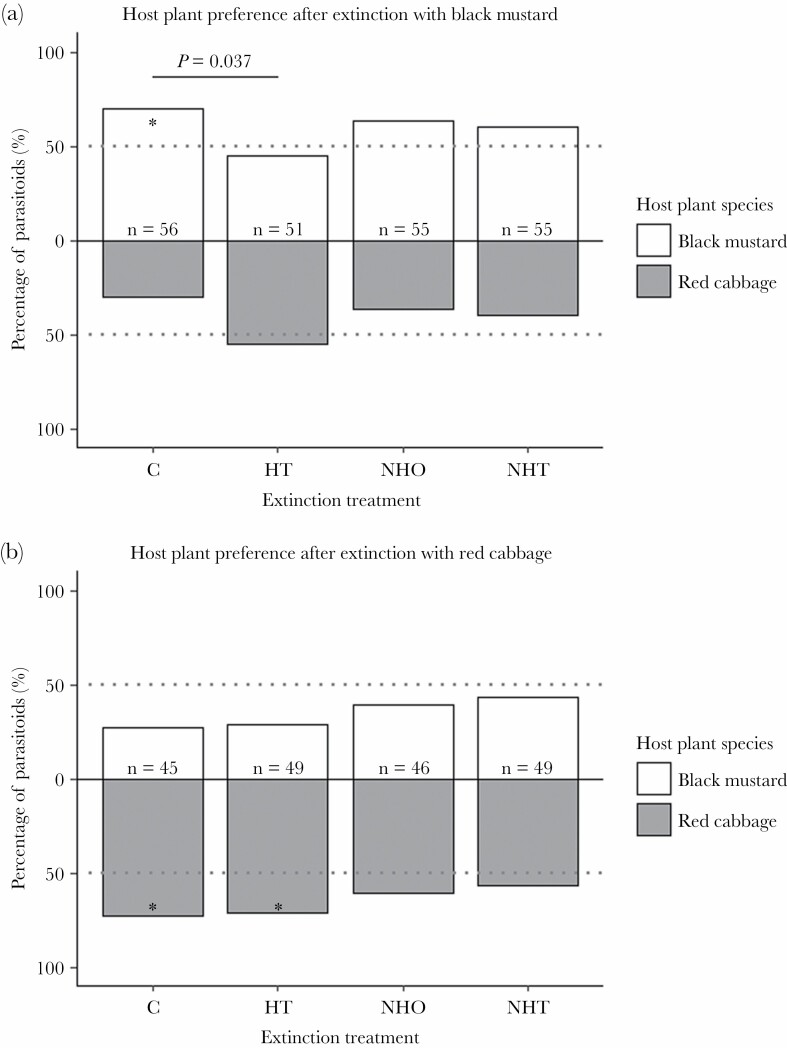
The plant volatile preference (%) for black mustard and red cabbage of the parasitoid *Cotesia glomerata* after parasitoids had been conditioned on either black mustard (a) or red cabbage (b) and subsequently experienced one of 4 extinction treatments. Parasitoids were assigned to no extinction experience (control, C), extinction with host traces (HT) or nonhost traces (NHT) for 15 min, or oviposition in a nonhost on a leaf with host traces, after which it was rested for 15 min in an empty Petri dish (NHO). A significant difference (GLMM, post hoc comparisons, *P* < 0.05) between the control (C) and any of the extinction groups is indicated with a line and a *P*-value above the two corresponding bars. Asterisks within bars indicate a significant preference (binomial tests, *P* < 0.05) for the respective plant species, and the *n* represents the sample size (total number of wasps tested more than 15 experimental days). The grey dotted line visualizes a deviation of the plant species preference from 50%.

We determined whether a conditioned preference for a particular plant species was influenced by an extinction experience 10 min after the rewarding experience. There was a marginally significant effect of the interaction between plant species and extinction treatment on parasitoid behavior (Figure 2, GLMM, $X_{3}^{2}=6.64$, *P* = 0.084), indicating that the effect of the extinction treatments may be different on the two plant species used for conditioning. We, therefore, present the results of the two plant species separately using results of the post hoc analysis.

Within treatment, binomial tests show that control groups of parasitoids conditioned on either black mustard or red cabbage have a clear preference for the conditioned plant species (Figure 2a, control red cabbage *P* = 0.002; control black mustard *P* = 0.005). When conditioning on black mustard was followed by any of the three extinction treatments, parasitoids had no significant preference for black mustard (Figure 2a, binomial tests, *P* = 0.576, *P* = 0.177, and *P* = 0.058 respectively). However, only the preference of parasitoids that had an extinction experience with host traces was significantly different from the preference of the control parasitoids (GLMM, control versus host traces, z.ratio = 2.676, *P* = 0.037, control versus nonhost traces, z.ratio = 1.097, *P* =0.692, control versus nonhost oviposition, z. ratio = 0.751, *P* = 0.876).

Parasitoids that were conditioned on red cabbage and subsequently had an extinction experience with nonhost traces or a nonhost oviposition, did not prefer volatiles of red cabbage over those of black mustard (Figure 2b, binomial tests, *P* = 0.392 and *P* = 0.184 respectively). In contrast, parasitoids still preferred red cabbage over black mustard after an extinction experience with host traces (binomial test, *P* = 0.004). The preference of the parasitoids of these three extinction treatments did not differ from those of the control treatment (GLMM post hoc, control versus host traces, z. ratio = 0.168, *P* = 0.998; control versus nonhost traces, z.ratio = 1.671, *P* = 0.339 and control versus nonhost oviposition, z.ratio = 1.206, *P* = 0.623), which shows that the three types of extinction experiences did not lead to a clear loss of the conditioned preference for red cabbage volatiles.

### Spontaneous recovery

In the extinction experiment, the only treatment in which a loss of the conditioned plant volatile preference and a difference in preference from the control group occurred was an extinction experience with host traces on black mustard. This treatment was therefore selected for the spontaneous recovery experiment. We found a significant effect of the interaction between extinction treatment and time point on parasitoid behavior in this experiment (GLMM, LRT = 3.95, d.f. = 1, *P* = 0.047), indicating that parasitoids of the extinction and control treatments behaved differently at the different time points. We therefore present results of the extinction and control group per time point.

**Figure 3:**
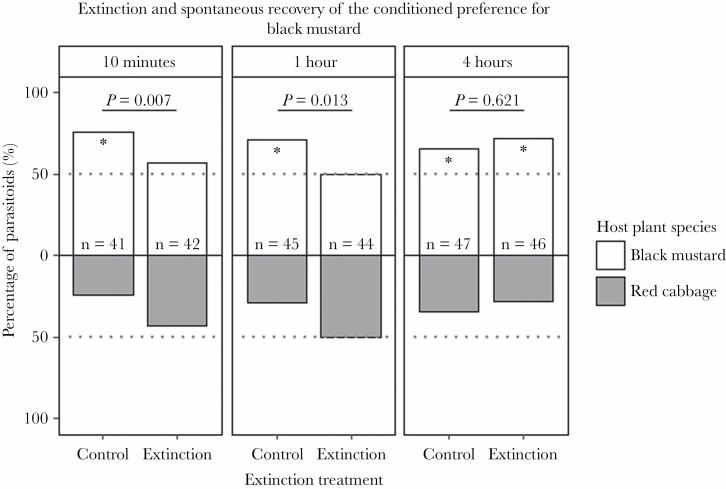
Extinction and spontaneous recovery of the conditioned preference for black mustard of *Cotesia glomerata* parasitoids after an extinction experience with host traces on black mustard. Parasitoids were conditioned on black mustard and then assigned to no extinction experience (Control) or a 15-min extinction experience with host traces (Extinction). Plant species preference was then tested at different time points; 10 min, 1 h and 4 h after the extinction experience, to check for extinction and subsequent spontaneous recovery of the black mustard plant volatile preference. *P*-values above bars indicate the statistical comparison of the control and extinction group at a specific time point. Asterisks within bars indicate a within-group significant preference (GLMM, post hoc comparisons, *P* < 0.05) for the respective plant species and the n represents the sample size. The grey dotted line was added to visualize a deviation of the plant volatile preference from 50%.

The control groups, where parasitoids were conditioned on black mustard and not given an extinction experience, maintained their preference for this plant species at each time point after conditioning (Figure 3, binomial tests, 10 min *P* = 0.009; 1 h *P* = 0.047; 4 h *P* = 0.029). The conditioned plant preference did not wane over this period of 4 h (GLMM control parasitoids, z.ratio = 0.961, *P* = 0.336). When an extinction experience with host traces occurred 10 min after conditioning, parasitoids did not have a preference for black mustard (binomial test, *P* = 0.860), and their preference was significantly different from that of the control parasitoids (GLMM post hoc, z.ratio = 2.679, *P* = 0.007). One hour after the extinction experience, *C. glomerata* still showed no preference for the conditioned black mustard plant (binomial test, *P* = 0.728), and compared with the control, this preference was still significantly different (GLMM post hoc, z.ratio = 2.489, *P* = 0.013). However, 4 h after the extinction experience, a significant preference for black mustard was found (binomial test, *P* = 0.017) and the conditioned preference had truly returned (GLMM post hoc, z.ratio = -0.495, *P* = 0.621). The fact that the conditioned preference is at first suppressed by the memory extinction treatment and later on reappears, together with the maintenance of the conditioned preference of the control group, shows that we observed spontaneous recovery of the conditioned plant volatile preference.

## DISCUSSION

Animals need to constantly adapt their behavior to efficiently locate essential resources in a dynamic environment (van Alphen et al. 2003; Eliassen et al. 2009). They can store information on local resource presence and distribution in their memory (Macphail 1996; Durier and Rivault 2000; Hoedjes et al. 2011), but this information needs to be reevaluated on each encounter of the resource-associated cues (Eisenhardt et al. 2013). In this study, we showed that a conditioned plant volatile preference in the parasitoid wasp *Cotesia glomerata* can disappear after an extinction experience, and that this conditioned preference can reappear over time, that is we observed spontaneous recovery of the conditioned behavior. Furthermore, we show that the consequences of an extinction experience can depend on the type of extinction experience as well as on the plant species.

*C. glomerata* has the ability to learn to associate herbivore-induced plant volatiles (HIPVs) with the presence of its host *Pieris brassicae* after a single oviposition in this host (Geervliet et al. 1998; Bleeker et al. 2006; Smid et al. 2007), which we have also shown here with black mustard and red cabbage. We subsequently tested if and how the resulting conditioned plant volatile preference was affected by different types of extinction experiences. We hypothesized that an extinction experience with host traces would not lead to the formation of extinction memory, because host traces may still be an indication of host presence and can lead to the formation of a reward memory (Meiners et al. 2003; Olson et al. 2003; Takasu and Lewis 2003; Hoedjes et al. 2011). However, parasitoids conditioned on black mustard lost their conditioned preference for this plant species after an extinction experience with host traces for 15 min. The conditioned plant volatile preference spontaneously recovered within 4 h, which indicates that the retrieval of the reward memory was temporarily blocked by the formation of extinction memory. Because associated HIPVs and host traces both indicate host presence, parasitoids may strongly anticipate host oviposition. Not meeting this anticipated reward within 15 min of host searching may have initiated the formation of the extinction memory, suggesting that alterations in the reliability of host-derived cues can trigger extinction memory formation.

To our initial surprise an extinction experience with nonhost traces also had the opposite effect of what we expected. We hypothesized that such an experience would trigger the formation of extinction memory, because encountering nonhost traces undermines the reliability of the association between the learned cues and the presence of hosts. However, when an extinction experience with nonhost traces of *Mamestra brassicae* occurred, the conditioned plant volatile preference of parasitoids did not change. These findings are in line with those described above for host traces, when considering that parasitoids likely do not anticipate a host nearby nonhost traces. Indeed, nonhost traces did not trigger the same excitatory behavioral response in *C. glomerata* as host traces in this study. When the reward expectation is low, a single 15-min extinction experience is apparently not sufficient to trigger the formation of extinction memory. In this case, the benefits of a change in behavior might not outweigh the costs of memory formation (Dukas 1999b).

An extinction experience with host traces on black mustard followed by oviposition in the nonhost *M. brassicae* also did not lead to a change in the conditioned plant volatile preference. This suggests that the oviposition in *M. brassicae*, which *C. glomerata* accepts for oviposition even though her offspring cannot survive in this nonhost (de Bruijn et al. 2018; de Bruijn et al. manuscript in preparation), may cancel out the effect of the encountered host traces. Though speculative, the parasitoid’s strong anticipation of a host oviposition on encountering host traces, may have been fulfilled by the nonhost oviposition. This finding may indicate that host-related extinction experiences might be more likely to alter foraging behavior than nonhost related extinction experiences, but this has to be studied further. Nevertheless, alterations in foraging behavior after a nonhost oviposition have also been reported, such as in Steven et al. (2019) who showed that unconditioned *Cotesia kariyai* parasitoids were willing to oviposit in nonhost caterpillars, but were subsequently less attracted to the nonhost infested plant. On the other hand, in a laboratory and a semifield study, conditioned *C. glomerata* parasitoids continued to visit nonhost infested plants, even after ovipositing in nonhosts (de Bruijn et al. 2018; de Bruijn et al., manuscript under revision). Such interactions with nonhosts and subsequent behavioral alterations are likely both parasitoid species-specific and nonhost-specific. Behavioral alteration in *C. glomerata* might require multiple nonhost (*M. brassicae*) encounters. Various studies, involving humans (Exton-McGuinness et al. 2015), rats (Harris and Andrew 2017), and insects (Lagasse et al. 2009) have indeed shown that the formation of an extinction memory depends on the number of extinction experiences, but whether this also applies to our study system remains to be investigated.

*Pieris brassicae* caterpillars occur on various brassicaceous plant species in nature (Bell and Muller 1973; Carter 1984), and we tested the effect of an extinction experience with two of them. Interestingly, we found a plant species-specific effect of an extinction experience with host traces. Parasitoids conditioned on black mustard lost their preference for this plant species after an extinction experience with host traces, although parasitoids conditioned on red cabbage did not. This host-plant specific effect suggests that conditioning on these plant species may result in different strengths of the association between the cue and reward. Both of these associations are strong enough to result in memory retention, yet differ in their sensitivity to an extinction experience. Further research could improve our understanding of the underlying mechanisms and causes. It is known that animals learn some associations more readily than others and that this is related to their ecology and evolutionary history (Dunlap and Stephens 2016; Smid and Vet 2016). This prepared learning is known to be host species-specific in *C. glomerata* (Kruidhof et al. 2012), but our findings indicate that it is also plant species-specific.

In conclusion, our study demonstrates extinction and spontaneous recovery of associated memory with cues and rewards that are relevant in an animal’s natural habitat, that is with HIPVs and a host oviposition reward in a parasitoid wasp. Though mechanistic studies on extinction and spontaneous recovery are ample, we have found only one comparable example for parasitoid wasps. In Papaj et al. (1994), the parasitoid *Leptopilina heterotoma* was conditioned on a host-infested mushroom substrate (defrosted grinded decaying mushroom) and an extinction experience on a mushroom substrate without hosts occurred 24 h later. The conditioned preference for the mushroom substrate disappeared and subsequently reappeared within 2 h. We expected that such fitness-related memories would be rather resistant to an extinction experience (Kaiser et al. 2003; Lagasse et al. 2009; Eisenhardt et al. 2013). Indeed, our results corroborate the findings of Papaj et al. (1994) and confirm that this is indeed the case because no permanent behavioral change was observed in *C. glomerata*. We did, however, find a temporary alteration of conditioned behavior. A single extinction experience with an uninfested substrate (Papaj et al. 1994) or host traces (this study) can be enough to temporarily suppress the retrieval of a host oviposition-related reward memory. This temporary suppression may be adaptive, when the lack of host finding accurately predicts current circumstances, but not future opportunities (Papaj et al. 1994). With the spontaneous recovery of the conditioned preference, parasitoids might still profit from a reward memory when environments continue to change.

Overall, we conclude that in *C. glomerata* an associative memory, formed with HIPVs and a host reward, can be more sensitive to host-related extinction experiences than nonhost related extinction experiences and that the effects of an extinction experience can be plant species-specific. A parasitoid’s response to an extinction experience seems to be shaped by its preparedness to learn, which is in turn shaped by its evolutionary history with host and host-plant related cues. To our knowledge, this is the first example of the effects of extinction experiences on foraging behavior using resources and cues that are naturally relevant to animal foraging behavior. These results, therefore, provide novel insights into how extinction experiences lead to changes in foraging behavior in an ecological context and further support the context-dependency of foraging behavior. They demonstrate that parasitoids employ a high degree of fine-tuning of (learned) behavior and we predict that these nuances in learned behavior also apply to other animals.

## FUNDING

This study was supported by Nederlandse Organisatie voor Wetenschappelijk Onderzoek open competition grant no. 824.14.023 to L.E.M.V.

## Data availability

Analyses reported in this article can be reproduced using the data provided by de Bruijn (2021).
